# Chemically-Modified Sepharose 6B Beads for Collection of Circulating Tumor Cells

**DOI:** 10.3390/biom13071071

**Published:** 2023-07-03

**Authors:** Haiyan Chen, Yiming Zhang, Xiaoxiao Ma, Bohao Zhou, Zhonghua Liu

**Affiliations:** 1 The National and Local Joint Engineering Laboratory of Animal Peptide Drug Development, College of Life Sciences, Hunan Normal University, Changsha 410081, China; 202220142900@hunnun.edu.cn (Y.Z.); 202170142750@hunnu.edu.cn (X.M.); zbh1195374211@hunnu.edu.cn (B.Z.); 2 East China Institute of Digital Medical Engineering, Shangrao 334000, China; 3 Department of Bioengineering, University of California-Berkeley, Berkeley, CA 94720, USA

**Keywords:** CTCs, Sepharose 6B, breast cancer, chemically modified

## Abstract

The isolation and quantitative characterization of circulating tumor cells (CTCs) are of great importance in cancer diagnosis and prognosis. However, isolating and detecting CTCs in whole blood presents a significant challenge due to the low numbers of CTCs (often ranging from one to five) in samples containing billions of erythrocytes. Recently, point-of-care devices that use antibody trapping coupled with remote immunofluorescence analyses have been described to identify the number and type of CTCs in blood. In this study, we propose a novel method for trapping and quantifying CTCs using Sepharose 6B beads of 45–160 μm size that are engineered with capture antibodies. Specifically, we employed CD44 antibody conjugates (bearing a maleimide group) that are specific to the CTCs of breast cancer to thiol-Sepharose beads 6B. These beads, when mixed with MDAMB231 and Jurkat cells and filtered through a 40 μm filter, can capture ~80% of MDAMB231 cells. Furthermore, the antibody-modified Sepharose 6B can be stored at four degrees Celsius for a period exceeding six months.

## 1. Introduction

Tumor circulating cells (CTCs) are defined as tumor cells that are shed spontaneously from the primary tumor or enter the peripheral blood circulation during the diagnosis and treatment processes [1]. The presence of CTCs in blood has been associated with clinical outcomes in several malignancies, such as breast [2], prostate [3], lung [4], stomach [5], and colorectal cancer [6]. These cells are known to contribute to cancer recurrence and metastasis in patients, making the detection of CTCs an important tool for early diagnosis, the rapid evaluation of chemotherapy drugs, individualized treatment including the clinical screening of drugs, the detection of drug resistance, the monitoring of tumor recurrence, and the development of new tumor drugs [7,8]. Therefore, the detection of CTCs has become a significant focus of clinical research.

Blood contains more than 10^9^ red blood cells, more than 10^6^ white blood cells, and more than 10^8^ platelets per milliliter, whereas the number of CTCs is only a few to several hundred per milliliter, making the separation and detection of CTCs from blood extremely challenging [9]. There are two main technologies used for CTC enrichment from whole blood, which are based on the physical and biological characteristics of tumor cells. The physical-based methods include density gradient centrifugation, filtration, cytolysis, the enrichment of cell deformability, enrichment of cell electrical characteristics, and ApoStream^TM^ technology [10,11]. Each method has its advantages and disadvantages. For example, density gradient centrifugation has a high recovery rate, is simple, economical, and practical, but has low sensitivity, poor repeatability, and high cost. On the other hand, ApoStream^TM^ is low-cost, high-efficiency, has high capture, and does not affect the subsequent culture in vitro. However, it lacks high specific tumor-associated antigen, is prone to cell loss, and is prone to aggregating in the magnetic field [12]. Currently, the most commonly used CTC enrichment method is the magnetic-free separation technique based on the biological characteristics of tumor cells. This method is highly specific, has a high enrichment rate, is sensitive, has a short reaction and binding time, is simple, and does not affect the biological activity or gene expression of cells. However, it is prone to aggregation in the magnetic field, which can affect cell morphology and activity. Furthermore, the separation of cells bound to magnetic beads is challenging, which can affect subsequent detection. The CellSearch system is used to quantify CTCs in peripheral blood and is currently the only test approved by the US Food and Drug Administration (FDA) for quantifying CTCs [13]. It effectively defines epithelial-derived CTCs as being positive for Ep-CAM, DAPI, and cytokeratin, and negative for CD45. Nevertheless, this method also produces false negatives. In 2013, a team at Massachusetts General Hospital developed a third-generation technology called CTC-iChip [14], which enables the sorting of rare CTCs from whole blood at a rate of 10^7^ cells per second. Dhar et al. developed an integrated microfluidic system that can concentrate rare cancer cells from 1 mL of whole blood to approximately 50,000 2 nL drops, composed of detection reagents, in 15 min [15]. Gruijs et al. developed a new method for detecting CTCs using the flow cytometer-based Attune NxT application, specifically designed to detect rare events in biological samples without the need for enrichment [16].

The enrichment, isolation, and identification of CTCs primarily rely on their biological characteristics, which highlights the crucial role of reliable biomarkers in ensuring detection sensitivity and specificity. However, due to the non-specific expression of normal cells and the occurrence of epithelial-to-mesenchymal transition (EMT), which can lead to a decrease in or loss of epithelial cell antigen expression and tumor heterogeneity, there is currently no ideal marker for CTC detection. The commonly used biomarkers for CTCs include epithelial markers such as cytokeratin (CK), epithelial cell adhesion molecule (Ep-CAM), and tumor-specific markers such as human epidermal growth factor receptor 2 (HER-2), human mammaglobin (h-MAM), and polymorphic epithelial mucin 1 (MUC-1). Typically, these methods use the anti-Ep-CAM antibody to trap CTCs, and post-labeling with anti-cytokeratin (CK) is used to confirm the identity of the CTCs [17]. CD44 has been widely used as a marker for CSCs in breast cancer and various other types of cancer [18]; in breast cancer cells, only some cells with the phenotype CD44^+^ have the ability to form tumor cells, and this type of cell can self-renew [19]. Moreover, some CTCs express mesenchymal markers such as vimentin [20], N-cadherin[21], and CD44[22], and there are also reports of CTCs expressing markers of both epithelial and mesenchymal lineage [23]. Additionally, studies of CTCs in primary and metastatic breast and prostate cancers have shown the presence of markers such as CD44C/CD24low, ALDH1, and Bmi1, which are associated with advanced disease and treatment resistance [24,25,26,27]. Pearl et al. described CD44 as a novel tumor progenitor marker for recognizing CTCs [28], highlighting its significance in the identification of CTCs. Importantly, the presence of CD44-positive CTCs consistently correlates with increased disease aggressiveness and an unfavorable prognosis in specific cancer types [29,30]. These findings underscore the potential utility of CD44 as a valuable biomarker for CTC detection and prognostic assessment in breast cancer.

The objective of this study is to develop a novel approach for detecting CTCs using Sepharose 6B beads, a type of commercial bead. These beads are characterized by a particle size ranging from 45 to 160 μm, making them easily detectable. We obtained thiol-containing Sepharose 6B beads via TCEP and chemically modified the antibody by introducing a maleimide group and a fluorescent label. The CD44 antibody was then connected to the Sepharose beads by a reaction between maleimide and thiol. Using these modified Sepharose 6B beads, we were able to capture approximately 80% of the cancer cells from a suspension. Importantly, the modified beads naturally settle without requiring centrifugation, and can thus be reused. Therefore, our approach presents a simple, efficient, and cost-effective method for detecting clinical CTCs.

## 2. Method

### 2.1. Synthesis of Antibody–Conjugate

The synthesis of the antibody–conjugate was performed as previously described [31]. To prepare the maleimide-anti-CD44-Cy5 (cyanine 5) conjugate (Cy5-CD44-MAL), CD44 antibody (5 mg/mL, 500 μL, Bio X cell, BE0262), 10 µL of 0.1 mol/L O-[N-(3-Maleimidopropionyl) aminoethyl]-O′-[3-(N-succinimidyloxy)-3-oxopropyl] heptacosa-ethylene glycol (NHS-PEG-MAL, Sigma, St. Louis, MO, USA), and 20 µL of 5 mg/mL Cy5 N-succinimidyl ester (Cy5-NHS, Lumiprobe, Cockeysville, MD, USA) in dimethyl sulfoxide (DMSO) were mixed and incubated in the dark for 2 h at 4 °C. The resulting product was purified using a PD-10 column (GE Healthcare Bio-Sciences, Piscataway, NI, USA, Sephadex G-25 M, GE17-0851-01) with 1× phosphate-buffered saline (PBS) pH 7.4 as the eluent, following the manufacturer’s instructions. The absorbance of the product was measured using UV–visible spectroscopy (UV/Vis (protein): λmax (ε) = 280 nm (210,000 M^−1^cm^−1^); UV/Vis (Cy5): λmax (ε) = 633 nm (250,000 M^−1^cm^−1^ in ethanol)).The concentration of the final product (maleimide-anti-CD44-Cy5) was determined by calculating the absorbance using the equation A = K×l×c (where A is the sample absorbance, K is the molar attenuation coefficient, l is the path length of the beam of light through the material sample, and c is the sample concentration). The purified conjugates were stored at −20 °C. To synthesize the TMR (5-carboxytetramethyl-rhodamine)-pan-keratin maleimide (TMR-pan-keratin-MAL), we followed the same method as described above, using pan-keratin antibody (Bio X cell) and 5-carboxytetramethyl-rhodamine N-succinimidyl ester (TMR-NHS, Lumiprobe, 17120).

### 2.2. Synthesis of Antibody–Conjugate Sepharose 6B Beads

To develop our novel method for detecting circulating tumor cells (CTCs), we utilized Sepharose 6B beads (GE Healthcare Bio-Sciences, 68517-67-9) with a mass of 2 mg and suspended them in 2 mL of ddH_2_O for 6 h at room temperature (R.T.); the hydrated beads were washed with ddH_2_O two times and resuspended with 500 µL of 10 mM Tris-HCl (pH 8.0). To activate the beads, we added 20 μL of 0.1 mol/L tris (2-carboxyethyl) phosphine (TCEP, Sigma, C4706) or 1, 4-Dithiothreitol (DTT, Sigma) and allowed them to incubate at R.T. for 1 h. After centrifuging the sample at 10,000 g for 2 min, we washed the beads three times with PBS. We then added Cy5-CD44-MAL to the TCEP-prepared Sepharose 6B bead solution and incubated it in the dark at R.T. for 1 h. Next, we washed the CD44-conjugate Sepharose 6B beads with PBS three times and stored them at 4 °C. We synthesized TMR-Pan-keratin-conjugate Sepharose 6B beads using the same method.

### 2.3. Kinetic Studies of Thiopropyl-Sepharose 6B Beads

To develop the method for detecting circulating tumor cells (CTCs), we prepared thiopropyl-Sepharose 6B beads by diluting 2 mg of the beads in 460 µL of 10 mmol/L pH 8.0 Tris-HCl and 20 µL of 0.1 mol/L TCEP solution, and incubating the mixture at R.T. for 1 h. We then added 20 µL of 1.5 mg/mL maleimide-anti-CD44-Cy5 (capture antibody) to the TCEP pre-treated thiopropyl-Sepharose 6B beads solution, and incubated the mixture at R.T. for 120 min. During this period, we monitored the Cy5 fluorescence intensity on beads (≥3 beads) using confocal laser scanning microscopy (CLSM) at different time points, namely 0, 0.5, 1, 2, 3, 4, 5, 6, 7, 8, 9, 10, 12, 14, 16, 18, 20, 22, 24, 26, 28, 30, 32, 34, 36, 38, 40, 42, 44, 46, 48, 50, 52, 54, 56, 58, 60, 65, 70, 75, 80, 85, 90, 110, and 120 min. We performed the kinetics experiments in triplicate to ensure accuracy.

### 2.4. Optimum Antibody Concentration

Maleimide-anti-CD44-Cy5 was synthesized as described above. To evaluate the optimal amount of conjugate needed for efficient binding to Sepharose 6B beads, varying amounts of the conjugate (2.5 µg, 5.0 µg, 10 µg, 20 µg, 30 µg, 40 µg, 50 µg, 60 µg) were incubated with 2 mg of TCEP-activated Sepharose 6B beads at RT for 1 h with shaking in the dark. After incubation, the Cy5 fluorescence intensity on the beads was measured using CLSM (Carl Zeiss LSM 700, Jena, Germany).

### 2.5. Cell Culture

MDAMB231 and Jurkat cells were obtained from the American Type Culture Collection (ATCC, Rockville, MD, USA) and cultured in DMEM (Gibco-Invitrogen, Waltham, MA, USA) supplemented with 10% (*v*/*v*) fetal bovine serum (FBS) (Gibco-Invitrogen) and 1% (*v*/*v*) antibiotics (100 units/mL penicillin and 100 μg/mL streptomycin). Cells were seeded on 100 mm culture dishes and incubated at 37 °C in a humidified atmosphere containing 5% CO_2_ until they reached 80% confluence; MDAMB231 cells were adherent and Jurakt cells were suspended. The cells were then collected for use in experiments.

### 2.6. CD44-Conjugated Sepharose 6B Beads Detected MDAMB231 Cells

MDAMBA231 cells and Jurkat cells were seeded on 100 mm culture dishes, and when cells reached 80% confluence, cells were collected. To label the cells, MDAMBA231 cells were stained with 5-Carboxyfluorescein diacetate (CFDA, Sigma, C4916), and Jurkat cells were stained with sulfo-Cyanin 3 NHS-Ester (Cy3-NHS, Lumiprobe, 11320) for 10 min. The dye-labeled cells were then washed with PBS three times to remove excess dye. Following that, the cells were fixed with 4% paraformaldehyde for 10 min and washed with PBS three times. For the capture experiments, 100, 1000, and 10,000 cells were added to 1 mL PBS solution or 1% FBS-containing medium containing CD44-conjugate Sepharose 6B beads and incubated for 2 h on a shaker in the dark. The capture efficiency was then detected using a CLSM (Carl Zeiss LSM 700, Germany) by counting the cells that were captured and the dissociated ones. For the specific experiments, 1 × 10^4^ MDAMB231 cells and 1 × 10^7^ Jurkat cells were added to 1 mL PBS containing CD44-conjugate Sepharose 6B beads and incubated for 2 h as described above. The dissociated cells were removed by passing the mixture through a 40 µm filter, and the captured cells were analyzed using a CLSM (Carl Zeiss LSM 700, Germany).

### 2.7. Stability Test of Cy5-CD44 Conjugate Sepharose 6B Beads, Cy5-CD44 Conjugate Sepharose 6B Beads-MDAMBA231 Cells

The preparation of Cy5-CD44 conjugate Sepharose 6B beads and the Cy5-CD44 conjugate Sepharose 6B bead-MDAMBA231 cells was performed as described above. The beads were stored at 4 °C, and their stability was assessed over a period of 28 weeks. The fluorescence intensity of the beads was measured using a CLSM (Carl Zeiss LSM 700, Germany) every two weeks, and pictures of the beads were taken. Additionally, the Cy5-CD44 conjugate Sepharose 6B beads were stored at 4 °C and tested for their ability to detect MDAMB231 cells, following the protocol described above.

### 2.8. Analysis

CLSM images were obtained using a Carl Zeiss LSM 700 instrument. The raw data were analyzed using Zeiss LSM ZEN 2009 software, GraphPad Prism 8.0 software, and the Image J program. To ensure consistency, all sample fluorescence intensities were measured and analyzed using the same CLSM parameters and Image J program. All the experiments were repeated more than three times.

## 3. Results

### 3.1. Overall Workflow

**Figure 1:**
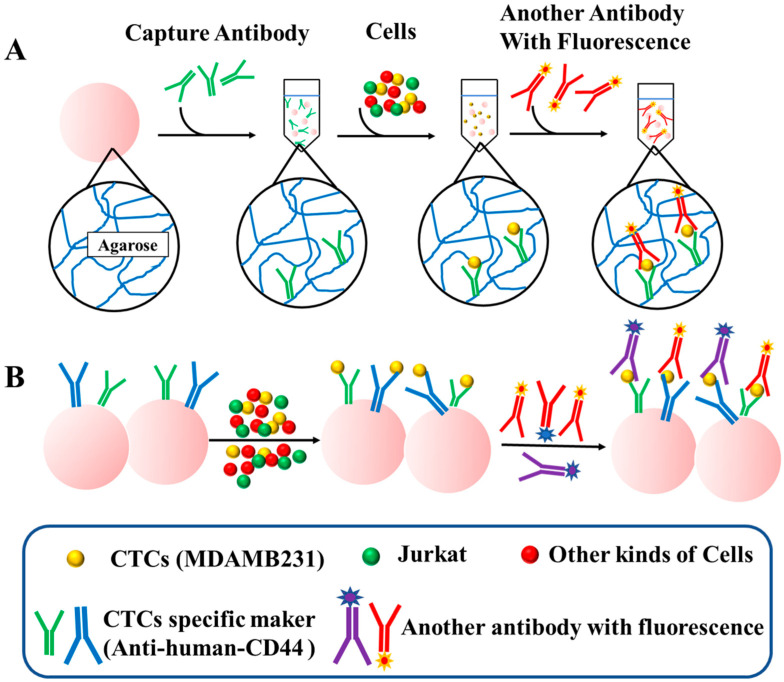
Overall workflow of chemically-modified Sepharose 6B beads for detecting CTCs. (**A**) Schematic diagram of the structure of the Sepharose 6B and the procedure of single marker detection. The Sepharose 6B beads are composed of agarose. The probe antibodies are immobilized on the Sepharose 6B by thiol groups of agaroses. (**B**) The procedure of multiplex detection based on the different kinds of antibodies.

Figure 1 provides a schematic representation of the workflow followed in this study. The first step involves the reaction of Sepharose 6B beads with modified antibodies possessing sulfhydryl groups. The beads are then incubated with a mixture of target cells and other cells to capture the target cells. After separation and purification, specifically bound cells are retained, while other cells are removed. CD44 is a well-known marker for CSCs in various types of cancer, including breast cancer [11]. In breast cancer cells, only a subset of cells expressing the CD44^+^ phenotype possess the ability to form tumors and self-renew [12]. Notably, it has been reported that CTCs also express CD44 [22,28], and the presence of CD44-positive CTCs consistently correlates with heightened disease aggressiveness and an unfavorable prognosis in specific cancer types [29,30]. Therefore, in this study, we employed CD44 antibodies to detect CTCs and chose the MDAMB231 cells with high CD44 expression as our target cells.

### 3.2. Features of Sepharose 6B Beads

**Figure 2:**
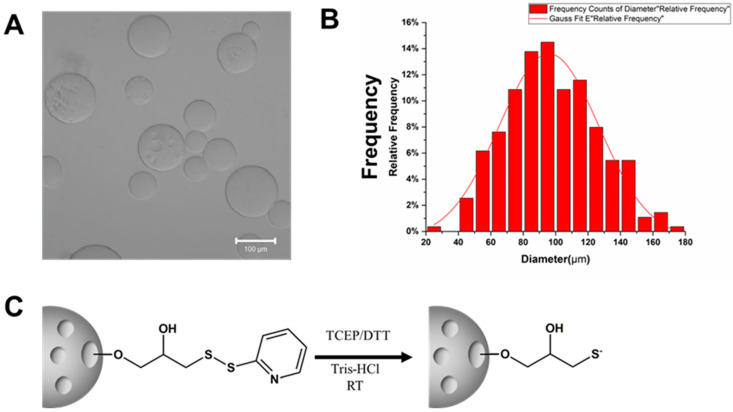
Features of Sepharose 6B. (**A**)Features of Sepharose 6B beads’ surface structure taken using a confocal microscope. Scale bar: 100 μm. (**B**) The size distribution of Sepharose 6B beads. (**C**) Through TCEP/DTT processing, the disulfide bond of the ball will be activated.

There are various types of beads available, such as Sephadex G-10, Sephacryl S200, Sepharose 4B, Sepharose 2B, and Sepharose 6B. Sepharose beads are composed of a cross-linked, porous agarose structure in bead form. The Sepharose 6B beads have a size range of 40–160 μm (Figure 2A,B), which makes them easy to separate and observe. To separate the beads, we used a 40 μm filter, which allowed the Sepharose 6B beads to remain in the upper layer while the free cells remained in the lower layer. The most significant advantage of Sepharose 6B beads is the presence of a disulfide bond that can be activated (reduced) by tris (2-carboxyethyl) phosphine (TCEP) or dithiothreitol (DTT) treatment, thus enabling further antibody binding (Figure 2C).

### 3.3. Sepharose 6B Beads Connected with Antibody

**Figure 3:**
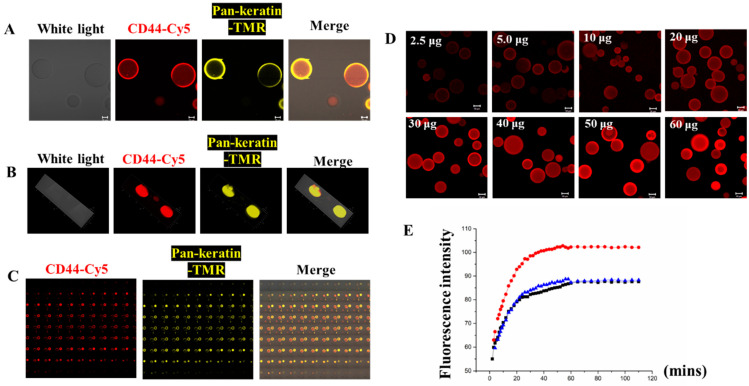
Sepharose 6B beads connected with antibodies. (**A**) The 2D images of Sepharose 6B beads coupled to CD44-Cy5 antibody and Pan-keratin-TMR, scale bar: 20 μm. (**B**) The 3D pictures of Sepharose 6B beads coupled to CD44-Cy5 antibody and Pan-keratin-TMR. (**C**) The 2D series pictures of Sepharose 6B beads coupled to CD44-Cy5 antibody and Pan-keratin-TMR. (**D**) Different amounts of CD44-Cy5 antibody connected to 2 mg Sepharose 6B beads. The appropriate amount of antibody is 30 μg, scale bar: 50 μm. (**E**) The kinetic study of fluorescence intensity changes with beads’ reactions over time. The blue, red and black lines represent three separate experiments of the kinetic study.

To conjugate the Sepharose 6B beads with CD44/Pan-keratin antibodies, we first prepared maleimide-anti-CD44-Cy5/maleimide-anti-Pan-keratin-TMR, which reacted with TCEP-activated thiopropyl-Sepharose 6B beads at room temperature. The coupling reaction was monitored and quantified by imaging the Cy5/TMR fluorescence of a small sample of Sepharose 6B beads with a confocal laser scanning microscope (Figure 3). Our kinetic experiments indicated that the beads were saturated with capture antibody conjugates within 60 min after mixing (Figure 3E), revealing that the optimal reaction time was 60 min. The uniform distribution of fluorescence in the beads indicated that the captured antibody conjugates were coupled throughout the three-dimensional matrix of the beads, and that the beads could couple with multiple antibodies at the same time (Figure 3A–C). Subsequently, we optimized the antibody concentration with 2 mg TCEP-activated thiopropyl-Sepharose 6B beads. The fluorescence imaging results showed that the Cy5 fluorescence intensity no longer increased after the antibody reached 30 μg, indicating that the reaction had become saturated (Figure 3D). Thus, our results suggest that the optimal reaction time for maleimide-anti-CD44-Cy5 with TCEP-activated thiopropyl-Sepharose 6B beads at room temperature is 60 min, and the optimal amount of antibody to react with 2 mg TCEP-activated thiopropyl-Sepharose 6B beads is 30 μg.

### 3.4. CD44-Antibody-Modified Sepharose 6B Beads in Detecting MADMB231 Cells

**Figure 4:**
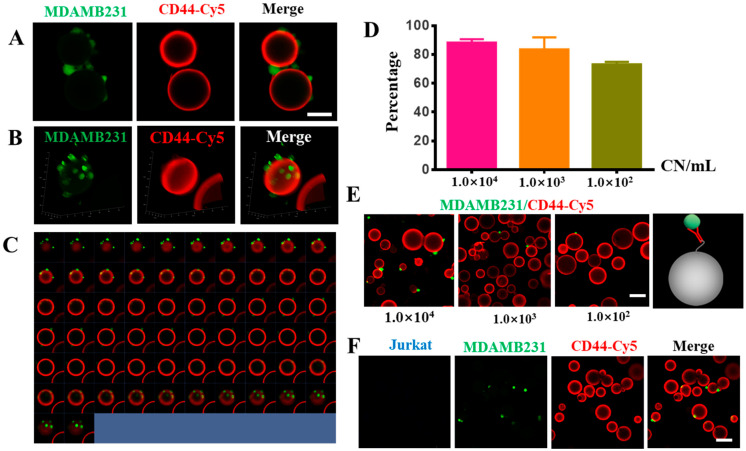
CD44-antibody-modified Sepharose 6B beads detected MDAMB231 cells. (**A**) The 2D images of CD44-antibody-modified Sepharose 6B beads after binding to MDAMB231 cells (green). Scale bar: 50 μm. (**B**) The 3D images of CD44-antibody-modified Sepharose 6B beads after binding to MDAMB231 cells (green). (**C**) The 2D series images of CD44-antibody-modified Sepharose 6B beads coupled to CD44 antibody after incubation with MDAMB231 cells. (**D**) The efficiency of CD44-antibody-modified Sepharose 6B beads in capturing MDAMB231 cells. Cell density of 10,000 cells/mL, 1000 cells/mL, 100 cells/mL. (**E**) The pictures of the cell capture efficiency of CD44-antibody-modified Sepharose 6B beads incubated with different concentration of MDAMB231 cells, scale bar: 100 μm. (**F**) The pictures of CD44-antibody-modified Sepharose 6B beads after incubation of MDAMB231 cells and Jurkat cells, scale bar: 100 μm.

To evaluate the efficiency of CD44-antibody-modified Sepharose 6B beads in capturing MADMB231 cells, we mixed different numbers of MDAMB231 cells with/without 1 × 10^7^ Jurkat cells in a 1 mL PBS solution, and added 1 mg of CD44 antibody-modified Sepharose 6B beads to the PBS solution to capture the MDAMB231 cells. The captured cells were collected through a 40 μm filter. Using this method, we were able to capture approximately 80% of MDAMB231 cells from the suspension, and the percentage of captured cells was dependent on the number of cancer cells (Figure 4D,E). The fluorescence imaging results demonstrated that MDAMB231 cells could bind to the surface of the CD44 antibody-modified Sepharose 6B beads (Figure 4A–C), while the Jurkat cells did not bind to the beads (Figure 4F), indicating the good specificity of this method. To simulate the blood sample environment, we detected MDAMB231 cells in 1% FBS-containing medium; the results showed that the CD44-modified Sepharose 6B could capture the MDAMB231 cells (Figure S1). Taken together, these results suggest that using specially modified antibody-coated Sepharose 6B beads for CTC detection is an effective and specific approach.

### 3.5. Antibody-Modified Sepharose 6B Beads Are Very Stable

**Figure 5:**
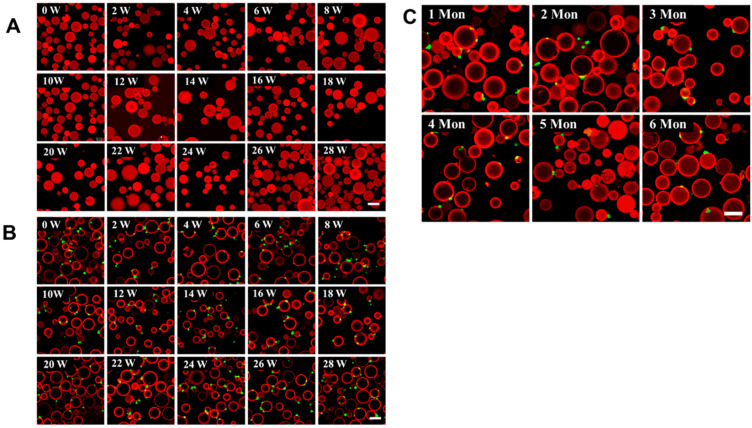
Assay of detected Sepharose 6B beads is very stable. (**A**) The fluorescence connected to Sepharose 6B beads is very stable for a long time, scale bar: 100 μm. (**B**) Cells captured by beads are stable for an extended period, scale bar: 100 μm. (**C**) The CD44-antibody-modified Sepharose is stable for 6 months and still works, scale bar: 100 μm.

The detection of CTCs is crucial for the early diagnosis and treatment of cancer. The ideal CTC detection method should be simple, highly efficient, specific, cost-effective, and stable [32]. To detect the stability of the antibody-modified Sepharose 6B beads, the beads and the beads with captured MDMB231 cells were kept in a 4-degree refrigerator for more than six months. The fluorescent image results showed that the intensity of antibody fluorescence was almost the same as in the newly modified case, even after 28 weeks (Figure 5A). What is more, the CD44-antibody-modified Sepharose 6B beads kept in a 4-degree refrigerator for more than six months could still capture MDAMB231 cells, and there was almost no difference in their ability to capture MDAMB231 cells (Figure 5C). We also evaluated the stability of the capture cells on the beads; the results showed that the MDAMB231 cells captured by the CD44-antibody-modified Sepharose 6B beads could be visible even when kept for 28 weeks in a 4-degree refrigerator (Figure 5B), which indicates that when we are very busy, there is no need to rush to perform the next detection as cells can be stored for a certain amount of time. It can also be used for the reconfirmation of the results, instead of repeating the detection. All these results suggest that the antibody-modified Sepharose 6B beads are a feasible method for the detection of CTCs.

## 4. Discussion

CTCs play a crucial role in linking in situ tumors with metastases, making the isolation and analysis of CTCs an important area of research for studying cancer genesis and metastasis [33,34]. CTCs are an essential marker in liquid biopsy and have a wide range of applications, including early tumor diagnosis, tumor progression evaluation, and efficacy monitoring [34,35,36]. However, detecting CTCs is a highly challenging task due to their scarcity [9]. Therefore, it is of great significance to develop more straightforward, cost-effective, reusable, and stable detection methods for CTCs.

In this study, we identified a commercially available Sepharose 6B bead that can be activated by TCEP or DTT and then chemically modified to conjugate a variety of antibodies. The resulting Sepharose 6B beads allowed for the even attachment of multiple antibodies to their surfaces, providing a valuable tool for using multiple markers to detect CTCs. At present, most CTC detection methods use the Ep-CAM antibody to capture and enrich CTCs using an immunomagnetic sphere, and they identify CTCs via CK19 antibody staining. The only FDA-approved CTC isolation assay, the CellSearch system, uses magnetic nanoparticles modified with anti-Ep-CAM antibodies to enrich CTCs [37]. However, not all tumors are derived from epithelial cells, and in epithelial–mesenchymal transformation (EMT), epithelial cells lose their epithelial phenotypes such as cell polarity and basement membrane junction, and gain the ability to migrate and invade, and thus tumor cells may no longer express Ep-CAM/CK [38,39]. Furthermore, aside from cancer, inflammation can also lead to the presence of epithelial cells in the bloodstream [40]. Thus, different CTCs may possess distinct biomarkers, necessitating the detection of specific biomarkers for each CTC subtype. Therefore, using multiple markers for detection can improve the sensitivity and specificity of CTC detection. This approach is advantageous in our method as it allows for the easy modification of Sepharose 6B with different antibodies tailored to specific CTCs from different types of cancer. We have demonstrated that CD44-modified Sepharose 6B beads can capture approximately 80% of MDAMB231 cells, and the percentage of captured cells is positively correlated with the number of cancer cells. The captured CTCs were easily collected by passing the suspension through a 40 μm filter, without requiring centrifugation. Subsequently, the CTCs could be released from the beads via trypsinization and used for further analysis without causing any significant loss in cell viability. These isolated CTCs can be cultured in vitro to obtain a large number of cells that can be tested for drug sensitivity, thereby facilitating the development of personalized treatment strategies for cancer patients [41]. Moreover, since the CD44-modified Sepharose 6B beads can be reused multiple times, this method could be cost-effective and practical for routine CTC detection and analysis.

Our findings are noteworthy as they demonstrate that the CD44-antibody-modified Sepharose 6B beads remained effective in capturing MDAMB231 cells even after being stored in a 4-degree refrigerator for over six months. The capture ability of MDAMB231 cells by these beads was comparable to that of newly modified beads. Furthermore, the captured cells remained stable on the beads for over 6 months, suggesting that the beads can be stored for an extended period without their efficacy being compromised. This stability is a crucial factor for clinical applications, as it allows for the reconfirmation of results, and eliminates the need for repeated detections when researchers are busy.

In this study, our objective was to maximize CTC capture efficiency by utilizing beads labeled with multiple antibodies against unique biomarkers to distinguish and quantify different types of CTCs present in blood samples. However, to detect the CTCs in blood in clinical situations is complex and subject to variations; therefore, the blood environment will also affect the capture efficiency. Therefore, for this method to be applicable in clinical settings, further experiments need to be conducted, such as evaluating the effectiveness of multiple antibodies connected to the Sepharose 6B beads for enriching different types of CTCs and testing this method on patient samples.

## Data Availability

Not applicable.

## Supplementary Materials

The following supporting information can be downloaded at: https://www.mdpi.com/article/10.3390/biom13071071/s1, Figure S1. 2D images of Sepharose 6B beads coupled to fluorescent antibodies directed against MDAMB231 biomarker in the 1% FBS containing medium, Scale bar: 100 μm.

## References

[1] Zhang X., Ju S., Wang X., Cong H. (2019). Advances in liquid biopsy using circulating tumor cells and circulating cell-free tumor DNA for detection and monitoring of breast cancer. Clin. Exp. Med..

[2] Cristofanilli M., Budd G.T., Ellis M.J., Stopeck A., Matera J., Miller M.C., Reuben J.M., Doyle G.V., Allard W.J., Terstappen L.W. (2004). Circulating tumor cells, disease progression, and survival in metastatic breast cancer. N. Engl. J. Med..

[3] de Bono J.S., Scher H.I., Montgomery R.B., Parker C., Miller M.C., Tissing H., Doyle G.V., Terstappen L.W., Pienta K.J., Raghavan D. (2008). Circulating tumor cells predict survival benefit from treatment in metastatic castration-resistant prostate cancer. Clin. Cancer Res..

[4] Tanaka F., Yoneda K., Kondo N., Hashimoto M., Takuwa T., Matsumoto S., Okumura Y., Rahman S., Tsubota N., Tsujimura T. (2009). Circulating tumor cell as a diagnostic marker in primary lung cancer. Clin. Cancer Res..

[5] Matsusaka S., Chin K., Ogura M., Suenaga M., Shinozaki E., Mishima Y., Terui Y., Mizunuma N., Hatake K. (2010). Circulating tumor cells as a surrogate marker for determining response to chemotherapy in patients with advanced gastric cancer. Cancer Sci..

[6] Huang X., Gao P., Song Y., Sun J., Chen X., Zhao J., Liu J., Xu H., Wang Z. (2014). Relationship between circulating tumor cells and tumor response in colorectal cancer patients treated with chemotherapy: A meta-analysis. BMC Cancer.

[7] Sawada T., Araki J., Yamashita T., Masubuchi M., Chiyoda T., Yunokawa M., Hoshi K., Tao S., Yamamura S., Yatsushiro S. (2016). Prognostic Impact of Circulating Tumor Cell Detected Using a Novel Fluidic Cell Microarray Chip System in Patients with Breast Cancer. EBioMedicine.

[8] Alunni-Fabbroni M., Sandri M.T. (2010). Circulating tumour cells in clinical practice: Methods of detection and possible characterization. Methods.

[9] Stoecklein N.H., Fischer J.C., Niederacher D., Terstappen L.W. (2016). Challenges for CTC-based liquid biopsies: Low CTC frequency and diagnostic leukapheresis as a potential solution. Expert Rev. Mol. Diagn..

[10] O’Shannessy D.J., Davis D.W., Anderes K., Somers E.B. (2016). Isolation of Circulating Tumor Cells from Multiple Epithelial Cancers with ApoStream((R)) for Detecting (or Monitoring) the Expression of Folate Receptor Alpha. Biomark Insights.

[11] Esmaeilsabzali H., Beischlag T.V., Cox M.E., Parameswaran A.M., Park E.J. (2013). Detection and isolation of circulating tumor cells: Principles and methods. Biotechnol. Adv..

[12] Gupta V., Jafferji I., Garza M., Melnikova V.O., Hasegawa D.K., Pethig R., Davis D.W. (2012). ApoStream(), a new dielectrophoretic device for antibody independent isolation and recovery of viable cancer cells from blood. Biomicrofluidics.

[13] Allard W.J., Matera J., Miller M.C., Repollet M., Connelly M.C., Rao C., Tibbe A.G., Uhr J.W., Terstappen L.W. (2004). Tumor cells circulate in the peripheral blood of all major carcinomas but not in healthy subjects or patients with nonmalignant diseases. Clin. Cancer Res..

[14] Ozkumur E., Shah A.M., Ciciliano J.C., Emmink B.L., Miyamoto D.T., Brachtel E., Yu M., Chen P.I., Morgan B., Trautwein J. (2013). Inertial focusing for tumor antigen-dependent and -independent sorting of rare circulating tumor cells. Sci. Transl. Med..

[15] Dhar M., Lam J.N., Walser T., Dubinett S.M., Rettig M.B., Di Carlo D. (2018). Functional profiling of circulating tumor cells with an integrated vortex capture and single-cell protease activity assay. Proc. Natl. Acad. Sci. USA.

[16] Gruijs M., Zeelen C., Hellingman T., Smit J., Borm F.J., Kazemier G., Dickhoff C., Bahce I., de Langen J., Smit E.F. (2022). Detection of Circulating Tumor Cells Using the Attune NxT. Int. J. Mol. Sci..

[17] Mostert B., Sleijfer S., Foekens J.A., Gratama J.W. (2009). Circulating tumor cells (CTCs): Detection methods and their clinical relevance in breast cancer. Cancer Treat. Rev..

[18] Dandawate P.R., Subramaniam D., Jensen R.A., Anant S. (2016). Targeting cancer stem cells and signaling pathways by phytochemicals: Novel approach for breast cancer therapy. Semin. Cancer Biol..

[19] Collina F., Di Bonito M., Li Bergolis V., De Laurentiis M., Vitagliano C., Cerrone M., Nuzzo F., Cantile M., Botti G. (2015). Prognostic Value of Cancer Stem Cells Markers in Triple-Negative Breast Cancer. Biomed. Res. Int..

[20] Kallergi G., Papadaki M.A., Politaki E., Mavroudis D., Georgoulias V., Agelaki S. (2011). Epithelial to mesenchymal transition markers expressed in circulating tumour cells of early and metastatic breast cancer patients. Breast Cancer Res..

[21] Balasubramanian P., Lang J.C., Jatana K.R., Miller B., Ozer E., Old M., Schuller D.E., Agrawal A., Teknos T.N., Summers T.A. (2012). Multiparameter analysis, including EMT markers, on negatively enriched blood samples from patients with squamous cell carcinoma of the head and neck. PLoS ONE.

[22] Theodoropoulos P.A., Polioudaki H., Agelaki S., Kallergi G., Saridaki Z., Mavroudis D., Georgoulias V. (2010). Circulating tumor cells with a putative stem cell phenotype in peripheral blood of patients with breast cancer. Cancer Lett..

[23] Armstrong A.J., Marengo M.S., Oltean S., Kemeny G., Bitting R.L., Turnbull J.D., Herold C.I., Marcom P.K., George D.J., Garcia-Blanco M.A. (2011). Circulating tumor cells from patients with advanced prostate and breast cancer display both epithelial and mesenchymal markers. Mol. Cancer Res..

[24] Alonso-Alconada L., Muinelo-Romay L., Madissoo K., Diaz-Lopez A., Krakstad C., Trovik J., Wik E., Hapangama D., Coenegrachts L., Cano A. (2014). Molecular profiling of circulating tumor cells links plasticity to the metastatic process in endometrial cancer. Mol. Cancer.

[25] Barriere G., Riouallon A., Renaudie J., Tartary M., Rigaud M. (2012). Mesenchymal and stemness circulating tumor cells in early breast cancer diagnosis. BMC Cancer.

[26] Tinhofer I., Saki M., Niehr F., Keilholz U., Budach V. (2014). Cancer stem cell characteristics of circulating tumor cells. Int. J. Radiat. Biol..

[27] Giordano A., Gao H., Anfossi S., Cohen E., Mego M., Lee B.N., Tin S., De Laurentiis M., Parker C.A., Alvarez R.H. (2012). Epithelial-mesenchymal transition and stem cell markers in patients with HER2-positive metastatic breast cancer. Mol. Cancer.

[28] Pearl M.L., Dong H., Tulley S., Zhao Q., Golightly M., Zucker S., Chen W.T. (2015). Treatment monitoring of patients with epithelial ovarian cancer using invasive circulating tumor cells (iCTCs). Gynecol. Oncol..

[29] Loreth D., Schuette M., Zinke J., Mohme M., Piffko A., Schneegans S., Stadler J., Janning M., Loges S., Joosse S.A. (2021). CD74 and CD44 Expression on CTCs in Cancer Patients with Brain Metastasis. Int. J. Mol. Sci..

[30] Wang Y.-W., Li L.-L., Lu M., Li H., Hu K.-W. (2022). Stem cell-like circulating tumor cells indicate poor prognosis in gastric cancer. Arch. Med. Sci..

[31] Gong Y., Marriott G. (2020). Bead-Based Immunocomplex Entrapment Assays for Rapid, Sensitive, and Multiplexed Detection of Disease Biomarkers with Minimal User Intervention. ACS Sens..

[32] Attard G., de Bono J.S. (2011). Utilizing circulating tumor cells: Challenges and pitfalls. Curr. Opin. Genet. Dev..

[33] Dive C., Brady G. (2017). SnapShot: Circulating Tumor Cells. Cell.

[34] Green B.J., Saberi Safaei T., Mepham A., Labib M., Mohamadi R.M., Kelley S.O. (2016). Beyond the Capture of Circulating Tumor Cells: Next-Generation Devices and Materials. Angew. Chem. Int. Ed. Engl..

[35] Heitzer E., Haque I.S., Roberts C.E.S., Speicher M.R. (2019). Current and future perspectives of liquid biopsies in genomics-driven oncology. Nat. Rev. Genet..

[36] Neoh K.H., Hassan A.A., Chen A., Sun Y., Liu P., Xu K.F., Wong A.S.T., Han R.P.S. (2018). Rethinking liquid biopsy: Microfluidic assays for mobile tumor cells in human body fluids. Biomaterials.

[37] Andree K.C., van Dalum G., Terstappen L.W. (2016). Challenges in circulating tumor cell detection by the CellSearch system. Mol. Oncol..

[38] Bulfoni M., Gerratana L., Del Ben F., Marzinotto S., Sorrentino M., Turetta M., Scoles G., Toffoletto B., Isola M., Beltrami C.A. (2016). In patients with metastatic breast cancer the identification of circulating tumor cells in epithelial-to-mesenchymal transition is associated with a poor prognosis. Breast Cancer Res..

[39] Hyun K.A., Koo G.B., Han H., Sohn J., Choi W., Kim S.I., Jung H.I., Kim Y.S. (2016). Epithelial-to-mesenchymal transition leads to loss of EpCAM and different physical properties in circulating tumor cells from metastatic breast cancer. Oncotarget.

[40] Cantaluppi V., Quercia A.D., Dellepiane S., Ferrario S., Camussi G., Biancone L. (2014). Interaction between systemic inflammation and renal tubular epithelial cells. Nephrol. Dial. Transpl..

[41] Sekine J., Luo S.C., Wang S., Zhu B., Tseng H.R., Yu H.H. (2011). Functionalized conducting polymer nanodots for enhanced cell capturing: The synergistic effect of capture agents and nanostructures. Adv. Mater..

