# Supplementary material for: Chemically-Modified Sepharose 6B Beads for Collection of Circulating Tumor Cells
Source: Biomolecules. 2023 Jul 3;13(7):1071. doi: 10.3390/biom13071071 (PMC10377418; doi:10.3390/biom13071071)
Supplement: Supplementary file 1 [file biomolecules-13-01071-s001.zip › biomolecules-2444464-supplementary.pdf]

Supplementary Materials:.

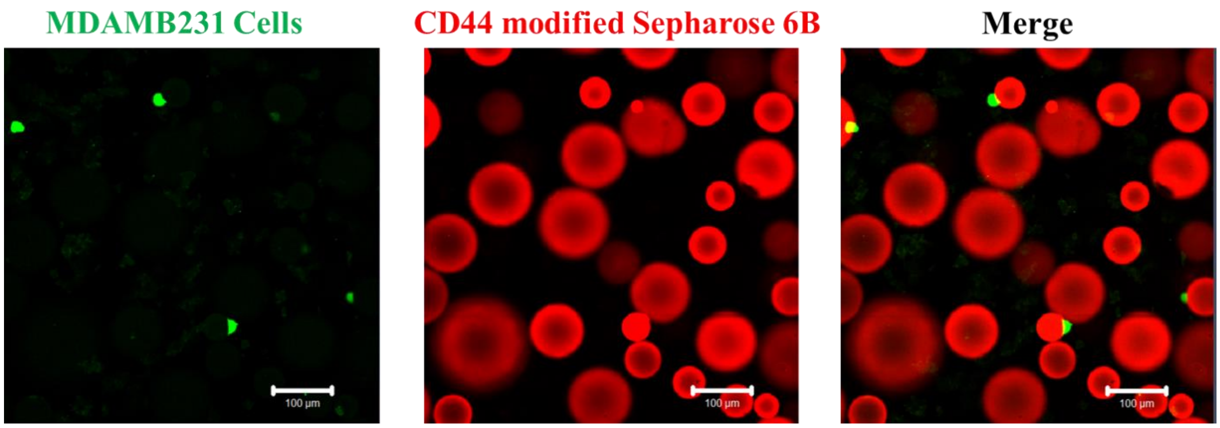

Figure S1. 2D images of Sepharose 6B beads coupled to fluorescent antibodies directed against MDAMB231 biomarker in the 1% FBS containing medium, Scale bar: 100 µm.
